# The Immediate Root-Filling Craze

**Published:** 1894-08

**Authors:** 


ARTICLE V.

### THE IMMEDIATE ROOT-FILLING CRAZE.

"Nothing" it is said, "is done without enthusiasm," and, in the remark, there is a great deal of truth, yet enthusiasts are apt to be just a little too sweeping in their assertions, a little too ready to set forth, as a dogma, what is at best an unproved statement. They draw conclusions from limited experience. Statistics supply them, as they will every one else, with the proofs they need, and fortified by these, they last but by no means least, look on those who differ from them, as ignorant, unskilled, foolish people doomed to oblivion, if not eternal damnation. Nevertheless, we owe these enthusiasts a great debt of gratitude, for though they may be at one extreme of the swing of the pendulum, yet it is they who have set it in motion, and the position of rest will, doubtless, be in advance of that which it aforetime held. It is scarcely needed to enumerate a list of points concerning which crazes have passed over the stage of dental practice. Crazes for a time, but prunned, modified, elaborated, they have taken their place, not ahead of and to the exclusion of other ideas and methods, but alongside these to be used in proper time and place. Nothing would prove our remarks better than the discussion on root-filling by the members of the Odontological Society, reported in our last issue. As is well known, the fashion of the day is to be an immediate root-filler. To be absolutely correct on this subject a man must be in a position to affirm, that he never dresses a tooth, fills them all at the first visit and never has a failure, or, if one should, by some malinterposition of Providence, occur, it is due to an occult cause, and that is of less potency, when brought beneath the magic influence of this special treatment, than when any other is tried. For dead teeth there is one treatment:—Immediate filling, and one drug, peroxide of hydrogen.

Broadly speaking dead teeth may exist in two conditions: in one the root membrane is healthy, in the other it is not. The extremes of either of these groups may be safely diagnosed, but those on the border land may give rise to difficulty and doubt. The first group indicates immediate filling, after clearing the pulp cavities. It is obvious, since beyond the apical foramen is simply healthy tissue, dressings, as far as this be concerned, would be injurious rather than otherwise. If the pulp cavities can be thoroughly cleansed, and for this copious syringing with water is as effectual as most other fluids, then dried and subsequently filled, micro-organisms, remaining in the tubuli, need be of little concern to us for we have abolished the conditions of their continued existence. Our second group of dead teeth form, however, the bone of contention over which we all love to have our little squabble. Seeing that dryness of pulp cavity, before filling, is one of the tenets of the immediate root fillers' creed, certainly a discharging tooth cannot be filled unless such discharge can be stopped at the sitting. This, we beg leave to state, cannot be done, save by the spurious means of plugging the apical foramen with some extraneous matter. It would be as rational to expect the discharge coming from a carious joint to cease because the surface openings had been washed with some patent cure-all. Many, who rank themselves with immediate fillers, recognise this fully, and treat such teeth by drilling into them and leaving them sc. Drainage, they undoubtedly obtain, and, if our existence were in an Utopia we could imagine such treatment perfect, unfortunately the vent, which forms the outlet, also forms the inlet, and to be logical, such a man should treat the above-mentioned carious joint in the same way, open it and leave it free to the winds oi Heaven and the dirt of earth. We certainly have never been able to appreciate the ground of such action, nor the reason why we should be called upon to abandon such dressings as common-sense and scientific teaching dictate.

Our question is, however, further complicated by, if not the majority, at any rate a great number of cases, in which there is no apparent discharge down the pulp cavity, but in which the root-membrane is obviously not healthy. Here, we would humbly suggest, the conditions of our work—would indicate the proper treatment, if the patient is coming, or can come again, it would be wiser to delay permanent work till then, if not, we must venture an immediate root filling, honestly recognising the fact, however, that we throw ourselves on the mercy of mother Nature, who, when we have removed the apparent cause of the disease, kindly overlooks our errors, and in spite, not because of them, completes the cure. And if some speaker should ask us the conundrum:—"If on the second visit the tooth had been found healthy why would it not have been advisable to have filled it on the first?" We would heave a sigh, that the days of prophecy are passed, and mildly suggest, that, if people could have forseen the awfully bad teeth they were destined to have, they would with their tiny fingers, have squashed their embyronic tooth germs in utero, and hate led a happy, if a toothless existence.—*Editorial in London Dental Record.*

